# Psychiatry

**Published:** 1904-05-21

**Authors:** 


### PSYCHIATRY.

**Mental Anxiety and Allied Conditions.**—The clinical study and analysis of anxious states in mental affections has in recent years disclosed a number of practical and interesting facts. Anxiety as a pathological manifestation, says Dr. Gaston Lalanne,[1] in a communication made at the Annual Congress of French Alienists and Neurologists recently held at Grenoble, is at first without an object (*sans object*), the patient feels anxious in spite of himself and without knowing why. There were few conditions so difficult to conceal as anxiety. As well in its slightest degree—uneasiness or restlessness—as in its greatest intensity, anguish, anxiety was a disturbance which was expressed by the entire being. The exciting causes were sometimes physical and sometimes psychical, and the symptoms manifested were both physical and mental. The physical symptoms comprised anæsthesias and hyperæsthesias of the organs of sense, cold feelings and chills of the scalp and body, horripilation, asthenia, and general lassitude, inco-ordination of voluntary movements far more apparent than real, emotional colouring of speech, and vertigo which was dependent upon vasomotor cerebral disturbances or upon digestive troubles. In the anxious states there were always circulatory troubles, such as accelerated heart-beat, irregularity of the heart's action, heightened arterial tension in association with constriction of the peripheral arterioles, and coldness of the extremities. Respiratory disturbances were also present in the form of acceleration and modification of the respiratory rhythm, even to the extent of dyspnœa, or the inspiration describedas "jerky" (saccadée) and mixed with sighs. Gastro-intestinal troubles were also present and included constipation, atony of the stomach and bowels, attacks of diarrhœa, and a variety of gastric pains. Finally, among the somatic troubles were to be noted those of secretory nature, such as polyuria, or occasionally retention of urine, salivation or on the contrary extreme dryness of the mouth, general or local sweating, or, on the other hand, extreme dryness of the skin. The psychical symptoms of "anxiety" include various degrees of vague dread and apprehensiveness, often taking definite forms, in which case they are designated as "phobias" or "obsessions of fear," weakening of the capacity of attention and of memory, and a tendency to confusion of ideas. Hallucinations of the senses are prone to occur in the confusional mental state just alluded to. The above physical and psychical troubles refer to mental states of anxiety in their pathological evolution, and are not intended to apply to physiological states of anxiety observed in normal individuals in certain circumstances of life, such as appearing at examinations, at public competitions, on the stage, etc. States of anxiety, and even of extreme anguish, may also attend such maladies as typhus, cholera, yellow fever, poisoning by carbon monoxide, by serpent venom or opium, angina pectoris, and pseudo-angina pectoris. Since the description of neurasthenia by Beard in 1883 much attention has been given to that ailment, and recent observers (Kaan, Hecker, and Freud) have recognised and separated from the general group of neurasthenia a special "neurosis of anguish"—a type of affection marked by general irritability with auditory hyperæsthesia, anxious attention, insomnia, and night terrors, attacks of anguish and distress, vertigo analogous to that which occurs in paralysis of the ocular muscles, feelings of dread, and "obsessions." The causation of this group of symptoms was traceable chiefly to sexual conditions, but other factors such as over-exertion and preoccupation, including night watching, were also present. Other authors (Pitres, Régis) while recognising this group of symptoms do not think that they constitute a special malady such as the term "neurosis of anguish" seems to imply.

**Anxious Psychopathies.**—Certain forms of insanity are met with in which the leading symptom during the period of development of the disease was anxiety and anguish. Among the accompanying clinical signs and symptoms, observed Lalanne,[2] were analgesias and hyperalgesias of the senses, disturbances of visceral sensibility, hallucinations which were visual or auditory, sometimes genital or verbal, hypochondriacal ideas and preoccupations relating to damnation, ruin, possession by evil spirits, etc., and occasional and sudden attacks of fright and terror. These conditions of anxiety with mental disturbance usually belonged to one of three categories, viz., simple melancholia without delusions, melancholia with præcordial anguish, and anxious or agitated melancholia. Melancholia with præcordial anguish formed the transition stage between the other two varieties. Other forms of mental disorder also existed in which anxiety was a prominent symptom. These included the insanity of degenerates, insanity of double form, the insanity of obsessions and impulses, alcoholic insanity and other psychoses from intoxication, and general paralysis of the insane. The epochs which were congenial to the evolution and growth of all forms of "insanity with anxious mental disturbances" were the periods of pubescence and adolescence between the ages of 15 and 30 years, the menopause, and the involution of old age. Heredity played a most important part in ætiology. Women seemed to suffer more often than men from the psychoses just alluded to. The prognosis of anxious mental states might be grave, adds Dr. Lalanne, according to the following criteria, viz., the tendency of the anxious state to persist, the presence or development of secondary mental disorder, such as obsessions of fear and doubt, and the tendency to alteration of the sense of personal identity. The treatment of the patient should be directed to improving the circulation, the muscular feebleness, and the condition of the brain and nervous system. Hence tepid and cold douches were useful, as were shower baths, and moderate physical exercise. Nitrite of amyl and nitroglycerine were useful in lowering arterial tension and in promoting the peripheral circulation, while the most useful cerebral sedatives to procure sleep and allay unrest were bromide of sodium, chloral hydrate, and opium in its various forms. Throughout the acute stage of mental anxiety and distress rest in bed should be enjoined, exercise and hydrotherapy being employed as the acute stage subsided.

**Epilepsy, Mental Automatism, and Crime.**—Dr. W. Spratling, medical superintendent of the Craig Colony for Epileptics, New York,[3] states that, although epilepsy is usually characterised by convulsions and loss of consciousness, convulsions or fits need not necessarily occur, and consciousness is not always lost, though it is always impaired or modified. The fit in its characteristic form constitutes one-half the picture of the disease, for the mental state following the fit forms the concluding half of the picture. Such post-epileptic mental states may present all varieties, from stupor and stupidity to a state of mental automatism in which criminal acts may be perpetrated. The insane epileptic in asylums has little facility for crime, partly because he is closely supervised, and partly because he is more or less demented and his power for evil thereby lessened. All epileptics do not eventually become insane. It is difficult to establish the exact proportion, but it may be said that not less than 20 per cent. of epileptics remain practically sane throughout their illness. An epileptic who is sane in the intervals between his fits may after a severe fit become so disordered in mind as to commit extraordinary crimes or misdeeds. In this condition consciousness is subverted or destroyed and the impulses to action which normally arise within the nervous system are deprived of all control. This subconscious state may last "from a few minutes to several days or even weeks, during which the patient is wholly automatic. He becomes a mere machine. He is able to go about as usual to take his meals and take care of his person, to dust and sweep his room and do sundry other commonplace things it was his habit to do before the attack. And yet he is all the while absolutely ignorant of what he is about, his mind is a blank, while such acts as the above go uninterruptedly on. In such a state he may do a criminal deed without criminal intent, as a railway engine may kill a person while running away without its driver." The same curious and interesting phenomena of automatism may appear independently in the absence of motor convulsions, *i.e.* in those attacks called "psychical epilepsy," which are characterised by automatism, or in cases of acute frenzied mental excitement lasting for a few hours or days. This psycho-epileptic frenzy is, adds Dr. Spratling, never momentary, as shown from a study of 1,300 cases. "I have not seen one of this type that lasted only a brief moment." The most difficult medico-legal problem connected with epilepsy is encountered in the purely psychical forms of epilepsy—those which are not complicated with convulsions, those which give no crude signs of their approach, which last for hours or days, or even weeks, and finally pass away as silently as they came. It is not safe, advises Dr. Spratling,[4] to oppose an epileptic while in the automatic state as he is then apt to become combative and dangerous. Thus a former patient of Craig Colony had an attack of *petit mal* while travelling on an elevated railway in New York. By the time the guard reached him he was all right, but when the time came for him to leave the car, some minutes later he made no move to go. The guard took him by the arm to move him towards the door when the patient struck at him violently and a rough and tumble fight ensued. He was then arrested by the police and locked up. "When he came to his senses, he was greatly mortified at being in gaol, and on showing his card of epileptic seizures was permitted to go." He was, says Dr. Spratling, unconscious when he struck the guard, had no intent whatever to do wrong, and was entirely blameless.

1 Revue Neurologique, Oct., 1902. 2 Loc. cit. 3 Medical Record, 1902. 4 Ibid.

*(To be continued.)*

